# Chemical Profile and Antioxidant Capacity of Propolis from *Tetragonula*, *Lepidotrigona*, *Lisotrigona* and *Homotrigona* Stingless Bee Species in Vietnam

**DOI:** 10.3390/molecules27227834

**Published:** 2022-11-14

**Authors:** Milena Popova, Boryana Trusheva, Ralitsa Chimshirova, Daniela Antonova, Kamelia Gechovska, Le Nguyen Thanh, Nguyen Thi Phuong Lien, Diep Thi Lan Phuong, Vassya Bankova

**Affiliations:** 1 Institute of Organic Chemistry with Centre of Phytochemistry, Bulgarian Academy of Sciences, Acad. G. Bonchev, Str., Bl. 9, 1113 Sofia, Bulgaria; 2 Institute of Marine Biochemistry, Vietnam Academy of Science and Technology, Hanoi 10000, Vietnam; 3 Graduate University of Science and Technology, Vietnam Academy of Science and Technology, Hanoi 10000, Vietnam; 4 Institute of Ecology and Biological Resources, Vietnam Academy of Science and Technology, Hanoi 10000, Vietnam; 5 Faculty of Natural Sciences, Quy Nhon University, Quy Nhon 55000, Vietnam

**Keywords:** stingless bees, propolis, chemical profile, antioxidant capacity, botanical sources, Vietnam

## Abstract

The present study aimed to analyze and compare the chemical profile and antioxidant capacity of propolis from different bee species and different regions. The chemical profiles of propolis from six stingless bee species (*Tetragonula iridipennis*, *T. laeviceps*, *Lepidotrigona terminata*, *L. ventralis*, *Lisotrigona carpenteri* and *Homotrigona apicalis*) collected from a total of eight locations in Vietnam were investigated by gas chromatography–mass spectrometry (GC-MS). More than 70 compounds were identified, amongst which phenolic lipids (cardanols, resorcinols and anacardic acids), aromatic acids, triterpenes and xanthones. Taxonomic markers for *Mangifera indica* (phenolic lipids and cycloartane triterpenes) were detected in propolis from bees of the genera *Tetragonula* and *Lepidotrigona*, although in different amounts, whereas propolis from *H. apicalis* was characterized by triterpenes of the amyrine type, typical of dipterocarp trees. A clear discrimination between both groups was observed by principal component analysis (PCA) and partial least squares–discriminant analysis (PLS-DA). Propolis from *Tetragonula* and *Lepidotrigona* spp. and from *Lisotrigona carpenteri*, which is rich in xanthones, possesses higher radical scavenging and ferric-reducing capacity than that from *H. apicalis*. Propolis produced by all six stingless bee species in Vietnam was analyzed for the first time. In addition, this is the first report on *L. carpenteri* propolis.

## 1. Introduction

Stingless bees, also called meliponines, are a large group of bees constituting the tribe Meliponini (family Apidae) [1,2]. More than 600 bee species have been described, distributed in subtropical and tropical regions [1,3,4]. Like the honey bee *Apis mellifera*, they are key pollinators in many ecosystems and produce high-value products such as honey and propolis [5,6].

Propolis is produced by bees after collecting plant resins and mixing them with small amounts of salivary gland secretions and wax. It is used by the bees to polish the internal walls of the hive and to seal cracks and crevices, and it serves as a basic material for the defense of the bee colony against parasites and predators [3,7]. Unlike *A. mellifera*, stingless bees use plant resins mixed with wax in nest construction, which is a material known as cerumen [7].

In nature, the nests of different stingless bee species can be found in hollowed cavities in tree trunks and branches and underground [3,4]. Nowadays, their farming is significantly increased, and dozens of different species are kept for honey and/or propolis (cerumen) production [2,4]. In addition, meliponiculture is of interest, as this practice improves household income, especially in the countryside and rural communities [4,6,8,9].

The healing properties of propolis have been recognized by humans since ancient times, and currently, it is well known to possess a wide spectrum of biological and pharmacological activities: antimicrobial, antifungal, antioxidant, antiviral, hepatoprotective, etc. [10,11,12]. Its potential to improve human health through the prevention and treatment of various diseases, such as laryngological, dermatological, gastrointestinal and cardiovascular disorders and coronavirus disease (COVID-19), was recently summarized [13]. Propolis activity is, however, related to the presence of different molecules, since its chemistry depends on the plants available around the beehive [13,14]. Various chemical compounds have been shown to be active principles, and they are more structurally diverse in propolis from tropical regions due to their rich plant biodiversity [15]. Nevertheless, bees, particularly the honey bee *A. mellifera*, including in temperate regions, have preferences for certain plant species that allow the formulation of propolis types based on the botanical source [14,15]. For stingless bee propolis, on the other hand, additional diversity and more complicated chemical composition were demonstrated, the reason for which might be related to the high diversity of stingless bees, as well as to their preferences for more than one plant in the same vicinity as a resin source [16,17]. To date, a few examples, particularly in the Indo-Malayan/Australasian region, have been shown for plants that have been visited by different bee species for propolis (cerumen) production, as recently reviewed [18]. Among them, the mangosteen (*Garcinia mangostana*; Clusiaceae) appears to be an attractive resin source for two stingless bee species in Thailand, and the mango tree (*Mangifera indica*; Anacardiaceae) is preferred by three bee species in different regions within Vietnam and Indonesia.

In this context and based on the insufficient data about the chemistry of stingless bee propolis, especially from South-East Asia, it is interesting to analyze and compare the chemical profiles and biological activities of samples collected from different bee species and different locations. In the present study, we report, for the first time, on the chemical profiles, antioxidant capacity and botanical sources of Vietnamese propolis produced by six bee species of the four genera *Tetragonula*, *Lepidotrigona*, *Lisotrigona* and *Homotrigona* collected from a total of eight locations. A clear discrimination of the samples from *Homotrigona apicalis* was found by chemometric methods, and dipterocarp trees were shown to be their botanical source. A preference for the mango tree was found for *Tetragonula* spp.: this is also one of the sources of propolis from *Lepidotrigona* bees. The bee *Lisotrigona carpenteri* seems to use only the resin of *Cratoxylum cochinchinense* (Hypericaceae) for propolis production. All studied samples displayed much lower antioxidant capacity than the honey bee *A. mellifera* propolis of the poplar type.

## 2. Results and Discussion

### 2.1. Chemical Profile of Stingless Bee Propolis

Propolis from six stingless bee species (*Tetragonula iridipennis*, *T. laeviceps*, *Lepidotrigona terminata*, *L. ventralis*, *Lisotrigona carpenteri* and *Homotrigona apicalis*) collected from a total of eight locations in Vietnam was studied (Table 1). The chemical profiles of the samples (25 samples; 70% ethanol extract) were analyzed by gas chromatography–mass spectrometry (GC–MS) after derivatization, which is one of the most frequently used techniques in propolis analysis [19]. More than 70 compounds belonging to various chemical classes were identified: sugars and sugar alcohols, fatty acids, aromatic acids, phenolic lipids, triterpenes, xanthones and tocotrienols (Table S1). The identified aromatic acids were benzoic, p-hydroxybenzoic, protocatechuic, vanillic and cinnamic acids, and the phenolic lipids were cardanols, resorcinols (cardols) and anacardic acids with alk(en)yl C13, C15, C17 and C19 side chains. Among the triterpenes, ketones, alcohols, aldehydes and acids of different skeletal types were detected: ursanes, oleananes, cycloartanes, damaranes and lupanes. All structural types of compounds have been previously found in stingless bee propolis, and except for xanthones, they are also known to exist in propolis from the honey bee *Apis mellifera*. For almost all samples, some of the compounds were partially identified, and only the structural type was shown based on the mass spectral fragmentation pattern.

In addition to sugars and fatty acids, propolis contains various secondary metabolites, which are commonly used as markers for plants that have been visited by bees for resin collection [14,19,20]. In line with this, many of the analyzed samples were characterized by the presence of cardanols, resorcinols and anacardic acids, which, together with triterpenes, mostly of the cycloartane type, such as cycloartenol, mangiferolic and isomangiferolic acids and ambolic acid, are a combination of compounds typical of the resin of the mango tree (*Mangifera indica*) [19]. The latter is a proven botanical source of propolis in different tropical regions for both stingless bees and honey bees [15,18]. Among the identified xanthones, in the highest relative amounts were α-mangostin, cochinchinone A and cochinchinone K, which, along with tocotrienols, are constituents of the resin from *Cratoxylum cochinchinense* [21,22]. This tree is widely distributed in Vietnam and has been previously shown to be a source of propolis from the stingless bees *Lisotrigona cacciae* and *L. furva* collected in Binhdinh province [17,23]. In some of the samples, dammarane, ursane and oleanane derivatives (ketones, aldehydes and acetates) were found, and their combinations are characteristic of dammar, a triterpene resin produced by trees belonging to the family Dipterocarpaceae [24,25]. Recently, *Shorea* spp. were suggested as probable dammar trees and one of the sources of Vietnamese propolis from *L. furva* [23]. Dammar trees are also a botanical source of Thai propolis from *Tetrigona melanoleuca* [26].

### 2.2. Chemometric Discrimination of Propolis Samples

**Figure 1:**
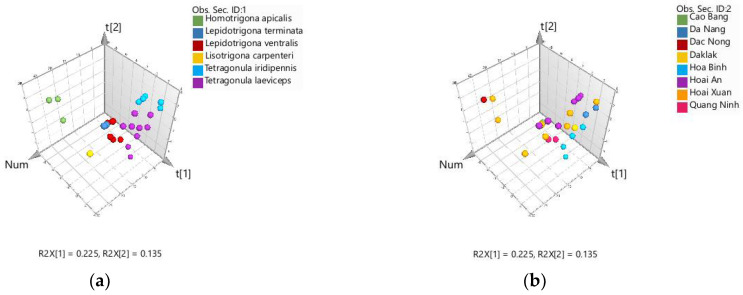
Three-dimensional PCA plots for 25 propolis samples: (**a**) colors correspond to the bee species; (**b**) colors correspond to the location of propolis collection.

Due to the large data set and in order to reveal whether there is any discrimination among the samples based on their chemical profiles with respect to their botanical source, chemometric methods were used. Principal component analysis (PCA) was first applied. PCA, as an unsupervised technique capable of the overall identification of metabolic differences, is a reasonable first step in a chemometric analysis [27]. In the analysis, except for triterpenes, semi-quantitative GC-MS data for the structural types of secondary metabolites (cardanols, resorcinols, anacardic acids, xanthones, aromatic acids and tocotrienols) were used, since some of these structural classes are characteristic of already-established plant species, as mentioned above. For the triterpenes, however, a different approach was chosen. The triterpenes were included in the analysis as individual compounds since, as a class of compounds, they are common for the resins of different plant species. Thus, the source of the triterpenes may be different, which primarily affects the structure and oxidation state of the individual compounds. The sugars and sugar alcohols and fatty acids were excluded from the analysis, as they are common propolis constituents. The PCA results are illustrated in Figure 1.

In the figure, two distinct groups can be seen, one of which consists of the three propolis samples of *H. apicalis* (V-15, V-17 and V-25) originating from two regions in Vietnam. In comparison with the other samples, they contained significant amounts of triterpenes and almost no aromatic derivatives. Moreover, the presence of compounds that were not detected in any of the samples from *Tetragonula*, *Lisotrigona* or *Lepidotrigona* spp., such as uvaol, erythrodiol, oleanolic aldehyde, ursolic acid acetate, oleanolic acid acetate and dammarenol, was found. All of these data show that the bee species, rather than the location, plays a role in the samples’ separation.

**Figure 2:**
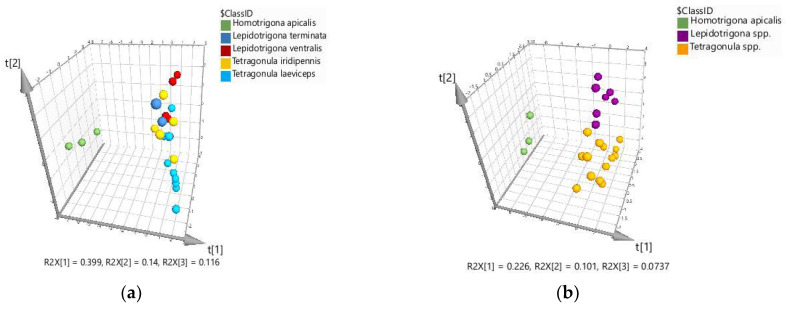
PLS-DA models for classification of 24 propolis samples based on 43 variables: (**a**) in accordance with five bee species (*Tetragonula iridipennis*—5 samples; *T. laeviceps*—9 samples; *Lepidotrigona ventralis*—5 samples; *L. terminata*—2 samples; *Homotrigona apicalis*—3 samples); (**b**) in accordance with three genera, *Tetragonula, Lepidotrigona* and *Homotrigona*.

Unlike *H. apicalis* propolis, however, propolis from the other stingless bee species is not well classified, although the tendency of discrimination could be presumed for *Tetragonula* and *Lepidotrigona* spp. (Figure 1a). So, to reveal and evaluate possible bee species’ preferences for a particular plant source of resins, a partial least squares–discriminant analysis (PLS-DA) was further applied. PLS-DA is a supervised method used to maximize and optimize the separation between groups of samples and consists of the separation of a priori given classes of objects [27,28]. As in PCA, the relative contents of groups of compounds and individual triterpenes were used as predictor variables. The PLS-DA score plot of the samples according to the bee species (predefined classes), except for the only sample of *L. carpenteri*, is presented in Figure 2a.

As expected, using PCA, propolis produced by *H. apicalis* was clearly discriminated, followed to some extent by that from *L. ventralis* and *L. terminata*. However, overlaps, especially between the samples from bee species of the genus *Tetragonula*, were observed. Based on these data, and having in mind that one of the species is represented only by two propolis samples, a new model was created with the bee genus instead of the bee species as predefined classes.

**Figure 3:**
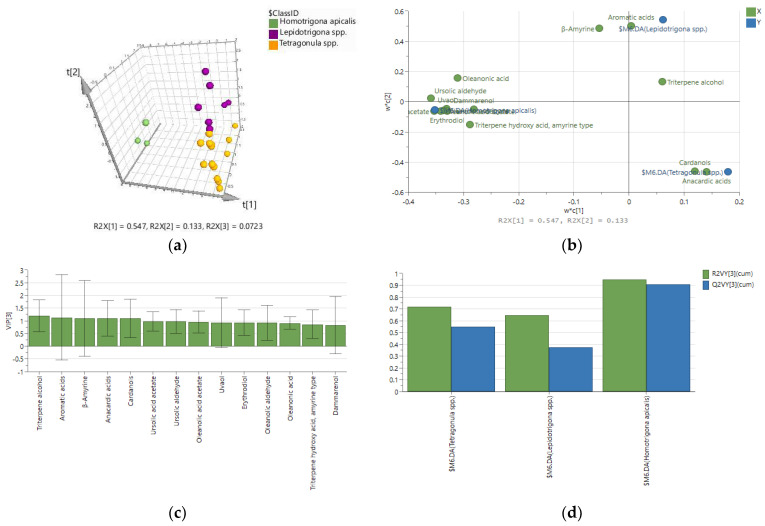
PLS-DA data for classification of 24 propolis samples based on 14 variables: (**a**) score plot; (**b**) loading plot; (**c**) variable importance in projection (VIP); (**d**) overall X/Y plot.

The score plot of the new PLS-DA model (Figure 2b) demonstrated a clear separation among the samples of the three stingless bee genera (R2X(cum) 0.400; R2Y(cum) 0.838; Q2(cum) 0.352), but the X variables were not well explained. Based on the loading plot and the variable’s contribution in the projection (VIP) score (Figure S1), 14 compounds/groups of compounds were distinguished as important for the samples’ classification. Using these marker compounds as predictor variables, a model with better performance was achieved (R2X(cum) 0.752; R2Y(cum) 0.771; Q2(cum) 0.666), presenting high model fitting (77.1%) and predictability (66.6%) (Figure 3). The overall X/Y plot demonstrated that the variables are moderately to highly explained and reliably predicted, with *Lepidotrigona* propolis having the lowest values. The PLS-DA model was further validated using a permutation test (Figure S2) and receiver operating characteristic (ROC) curves (Figure S3). The misclassification table shows that 95.83% of the samples were correctly classified (Table S2); only one sample of *Lepidotrigona* spp. was predicted as propolis from *Tetragonula* spp.

In the final model, eight variables (aromatic acids, cardanols, anacardic acids, *β*-amyrin, ursolic aldehyde, ursolic and oleanolic acid acetates, and one unidentified triterpene alcohol) were the most responsible for the discrimination of the samples. Among them, the compounds ursolic aldehyde and ursolic and oleanolic acid acetates, characteristic of dipterocarp trees, contributed to the clearest classification of *H. apicalis* propolis, and even based only on three samples, it could be assumed that the bee *H. apicalis* prefers dammar trees as a resin source. Moreover, none of the *H. apicalis* samples contained, even in trace amounts, markers for the mango tree, which is the source/one of the sources of propolis collected from *Tetragonula* and *Lepidotrigona* spp. (Table 1). *Tetragonula* bees showed a preference for *M. indica*, as the propolis they produced was distinguishable in cardanols and anacardic acids. In almost all samples, high amounts of recorcinols and cycloartane triterpenes were also detected (Table S1). Propolis from bees of the genus *Lepidotrigona*, however, displayed a more complicated and diverse chemical composition when compared with the other stingless bees, which is, in fact, the reason for the lowest predictability for this group of samples (Figure 3d). Along with the mango tree, *Lepidotrigona* spp. seem to choose one or more other plants for resin collection as well, which remained unidentified. All samples contained aromatic acids, and their relative amounts, especially benzoic, protocatechuic and vanillic acids, were the highest in two of the samples: V-21 (*L. ventralis*) and V-24 (*L. ventralis*). Distinguishable amounts of *β*-amyrin were also noticed, mainly in V-16 (*L. terminata*), V-21 (*L. ventralis)* and V-23 (*L. terminata*) (Figure 3b; Table S1).

Additionally, it should be mentioned that propolis from several *Lepidotrigona* and *Tetragonula* bee species collected in different regions in Vietnam contained xanthones typical of the resin from *C. cochinchinense*. Their relative contents ranged from 1.5 to 38.6% of total ion current (TIC); in samples V-14 (*L. ventralis*) and V-22 (*T. laeviceps*), they were among the major constituents. These data indicate that some of the propolis samples from *Lepidotrigona* bees, for example, are at least of triple-plant origin, which may be related to the assumption that resins of different plant species may benefit stingless bees as a barrier against different predators and pathogens [7,16]. Propolis from *Lisotrigona carpenteri* (V-10), analyzed for the first time, was characterized by xanthones, mainly cochinchinone A and *α*-mangostin, as the dominating group of constituents. Xanthones were previously described as propolis constituents in samples collected from beehives of *T. laeviceps*, *L. terminata* and *L. ventralis* in Thailand [26,29]. Their plant origin, however, was attributed to *Garcinia mangostana* (Clusiaceae) [26,30], which contains prenylated xanthones instead of the combination of prenyl and geranyl(oxy) derivatives characteristic of the resins of *C. cochinchinense*.

All presented data show that the stingless bee species plays some role in the choice of certain plants as sources of resin, and this role seems much more pronounced at the genus level. The results are somewhat supported by the fact that the stingless bee *H. apicalis*, for example, has been documented to visit dipterocarp trees and to use copious amounts of tree resin in nest building [31,32]. In addition, a chemical profile somewhat similar to that of *H. apicalis* propolis was shown for *Tetrigona appicalis* (Smith, 1857), the synonym of *H. apicalis* from Malaysia [33]. Mohamed et al. [33] reported high amounts of *α*- and *β*-amyrins and sesquiterpenoids in underivatized 80% ethanol extract. Diverse minor compounds corresponding to sesquiterpenoid molecules (M^+^, *m*/*z* 202–234; RT 28–35 min; Figure S4) were also observed in the GC chromatograms of all three studied *Homotrigona* propolis samples. However, they remained unidentified, because a different approach should be applied for their isolation and reliable identification, which was not the goal at this stage of research. Dipterocarp trees are one of the preferred resin sources of stingless bees in Borneo (Malaysia), as shown by behavioral assays and chemical similarities between the terpene (sesqui- and triterpene) chemical profiles of stingless bees and dipterocarp trees’ resins. [34,35]. Despite the wide distribution of dipterocarp trees in Vietnam [36], they do not seem to be attractive to the studied *Tetragonula* and *Lepidotrigona* spp. and *Lisotrigona carpenteri*. GC chromatograms of *L. carpenteri* propolis and selected samples from *Tetragonula* spp., *Lepidotrigona* spp. and *H. apicalis* are presented in Figure S4.

### 2.3. Antioxidant Capacity of Stingless Bee Propolis

Further, the antioxidant capacity of the samples was investigated in vitro by 2,2-diphenyl-1-picrylhydrazyl (DPPH) and ferric reducing antioxidant power (FRAP) assays. These methods, based on electron transfer reactions resulting in the reduction of a colored 2,2-diphenyl-1-picrylhydrazyl radical and ferric ion (Fe^3+^), respectively, have been widely applied in natural product science [37]. The obtained results are presented in Table 2. Strong positive and significant Pearson’s correlations between the values of the antioxidant capacity obtained by both methods were found (*r* = 0.8835, *p* < 0.00001; *r*^2^ = 0.7806 at *p* < 0.01 significance level), which reveals the suitability and reliability of the used methods [38].

The antioxidant properties of the extracts varied significantly from 2.45 to 66.83% inhibition in the DPPH assay and from 103.58 to 1156.47 μmol/L in the FRAP assay, and all of the extracts were less effective than the extract of Bulgarian propolis, which was used as a standard. Bulgarian propolis, which is rich in phenolic acids, phenolic acid esters and flavonoids [39], is a typical representative of honey bee propolis of poplar origin. Poplar-type propolis is one of the most studied ones, and its antioxidant power, due to the high amounts of phenolic constituents, is well documented [40,41]. In general, studied stingless bee propolis from *Tetragonula* and *Lepidotrigona* spp. displayed higher antioxidant properties when compared with propolis from *H. apicalis.* For the ferric reducing power, statistically significant differences were observed between samples of the genera *Lepidotrigona* and *Homotrigona* (*p* = 0.0200) and between *Lepidotrigona* and *Tetragonula* (*p* = 0.0114) in Tukey’s HSD test, which may be due mainly to the propolis from *L. ventralis*. The latter exhibited statistically higher reducing power in comparison with *H. apicalis* (*p* = 0.0072), *T. laeviceps* (*p* = 0.0204) and *T. iridipennis* (*p* = 0.0023) propolis. For radical scavenging ability, differences between *L. ventralis* and *H. apicalis* (*p* = 0.0395) and *T. iridipennis* (*p* = 0.0157) were found. Amongst the individual samples, the ethanolic extracts of V-7, V-21 and V-24, all collected from *L. ventralis* but from different regions, showed the highest antioxidant capacity, as measured by the DPPH and FRAP assays. High potential was also found for samples V-4 and V-5, both collected from *T. laeviceps*.

The obtained results are in good accordance with the fact that the propolis samples from all *Tetragonula* and *Lepidotrigona* bee species contained phenolic compounds, although in different relative amounts. Having in mind that the phenolic compounds are well known as antioxidants [37], a Pearson’s correlation analysis was applied between the relative amounts of each group of compounds (phenolic acids, cardanols, resorcinols, anacardic acids and xanthones and their combination) and DPPH and FRAP values (Table S3). As a result, moderate positive correlations, significant at a level of *p* < 0.01/0.05, were found between the relative contents of phenolic acids and xanthones and their sum and the DPPH values, as well as between the sum of phenolic acids and xanthones and the FRAP values. For phenolic acids, a strong correlation was detected with the FRAP values. The data indicate that the phenolic acids and xanthones are major groups of compounds contributing to the radical scavenging activity and ferric-ion-reducing power of the propolis, while phenolic lipids do not add to the antioxidant capacity of the propolis. Similar results were obtained by Kubo et al. [42] and Masuoka et al. [43], who found no DPPH scavenging activity for anacardic acids, cardanols or resorcinols (cardols). These compounds were shown to act as antioxidants in other ways, including the inhibition of xanthine oxidase and metal chelation, where the activity was mainly associated with the alkenyl side chain [42,44,45]. Among the studied stingless bee species, only the antioxidant ability of *L. terminata* propolis from Malaysia was shown [46]. Although the radical scavenging activity against DPPH radicals was tested, no reliable comparison can be made due to the lack of sufficient chemical data and the different test procedure used.

## 3. Materials and Methods

### 3.1. Propolis Samples

The bee species, geographical location and time of collection of propolis samples are listed in Table 1. The stingless bee species were identified by Dr. Nguyen Thi Phuong Lien, Department of Insect Ecology, Institute of Ecology and Biological Resources, Vietnam Academy of Science and Technology. Bulgarian propolis collected from honey bee hives in the area of the town of Elena, Balkan Mountains, was used as a standard in the evaluation of the antioxidant capacity of stingless bee propolis.

### 3.2. Extraction of Propolis

Propolis, grated after cooling, was extracted twice for 24 h each with 70% ethanol (1:10, *w*/*v*) at room temperature. The extracts of each sample were combined and evaporated in vacuo. The dry extracts, one for each sample, were used as the initial material for the evaluation of their chemical composition and antioxidant capacity.

### 3.3. GC-MS Analysis and Compound Identification

A gas chromatography–mass spectrometry (GC-MS) analysis was performed as previously described [17]. About 5 mg of the dry residue was mixed with 50 μL of dry pyridine and 75 μL of N,O-bis(trimethylsilyl)trifluoracetamide (BSTFA) and heated at 80 °C for 20 min. Reference compounds, previously isolated from Vietnamese stingless bee propolis [23], were subjected to the same silylation procedure, as about 1 mg of the compound was mixed with 10 μL of dry pyridine and 15 μL of BSTFA. The GC-MS analysis of the silylated extracts and reference compounds was performed on an Agilent 7820A GC System Plus gas chromatograph coupled with a 5977B Mass Selective detector and flame ionization detector (Agilent Technologies, Palo Alto, CA, USA) equipped with mid-polar DB-17HT (J&W Scientific, Folsom, CA, USA; 60 m long, 0.25 mm i.d. and 0.15 μm film thickness capillary column). The temperature was programmed from 75 to 325 °C at a rate of 5 °C/min, with a 20 min hold at 325 °C. Helium was used as a carrier gas at a flow rate of 0.8 mL/min; the split ratio was 1:50. Electron ionization (EI) mode at 70 eV was used; the ion source and quadrupole temperatures were set to 230 °C and 150 °C, respectively. The mass scan range was 45–1050 *m*/*z*. Instrument control and data collection: Mass Hunter Workstation Software (Revision B.06.07, Agilent Technologies). The ion current generated depends on the characteristics of the compound concerned, and it is not a true quantitation. The semi-quantification of the compounds is based on internal normalization with the area of each compound; the percentage of the individual compounds refers to the percent of the Total Ion Current (TIC) [19]. The identification of the compounds was performed using commercial libraries (NIST 14, Wiley 7th Mass spectra register), literature data and/or comparison with mass spectra of the reference compounds. In cases when identical spectra were not found, only the structural type of the corresponding component was proposed on the basis of its mass spectral fragmentation pattern.

### 3.4. Free Radical Scavenging Activity

The procedure for determining the scavenging activity of the extracts against the 2,2-diphenyl-1-picrylhydrazyl (DPPH) free radical (RSA) was performed as previously described [47]. Each Vietnamese propolis extract (5 mg) was diluted to 5 mL with MeOH (final concentration 1 mg/mL), and for Bulgarian propolis, used as a standard, 2.5 mg was diluted to 25 mL with MeOH (final concentration 0.1 mg/mL). Then, 2 mL of fresh methanolic DPPH solution (0.1 mM) was mixed with a 100 µL aliquot of each tested sample. After 30 min storage in a dark place, the decrease in the absorption was measured at 517 nm using a UV-vis spectrophotometer (Thermo Scientific Helios gamma). The results were expressed as percentages with respect to the control value. The DPPH activity of the tested samples was calculated by the following equation:

RSA (%) = [(A_0_ − A_S_)/A_0_] × 100 (1)

where A_0_ is the absorbance of the control sample (100 µL of MeOH instead of the aliquot volume of the sample was added), and A_S_ is the absorbance of the tested sample. Every sample was analyzed in triplicate.

### 3.5. Ferric Reducing Antioxidant Power (FRAP) Assay

In the FRAP assay, the ferric ion complexed with 2,4,6-tri(2-pyridyl)-1,3,5-triazine (TPTZ) acts as both an oxidant and a chromophore. The reaction of the Fe^3+^-(TPTZ)_2_ complex with an antioxidant generates the reduced form, Fe^2+^-(TPTZ)_2_, which absorbs light at around 600 nm [48]. The assay was performed as published before [49], with slight modifications. The FRAP reagent was freshly prepared by mixing ten parts of 0.3 M acetate buffer (pH 3.6), one part of TPTZ in 40 mM HCl and one part of 20 mM FeCl_3_·6H_2_O in distilled H_2_O. The reaction was started when the FRAP reagent (3 mL) was mixed with the tested sample (100 µL of a solution with a concentration of 1 mg/mL or 0.1 mg/mL in MeOH for Vietnamese and Bulgarian propolis, respectively). After 30 min at room temperature in darkness, the absorbance was measured at 593 nm against a blank. The FRAP value, expressed as µmol Fe^2+^/L, was calculated from a calibration curve of FeSO_4_·7H_2_O standard solutions.

### 3.6. Chemometric and Statistical Analyses

SIMCA software (version 17.0.2., Umetrics, Umeå, Sweden) was used for principal component analysis (PCA) and partial least squares–discriminant analysis (PLS-DA). The values of DPPH and FRAP were represented as means ± standard deviation of triple replicates. Pearson’s correlation, one-way ANOVA and Tukey’s post hoc test at significance levels of *p* < 0.01 and/or *p* < 0.05 were performed with Excel (ChemOffice 2019).

## 4. Conclusions

The chemical composition and antioxidant capacity of Vietnamese propolis produced by six stingless bee species of the four genera *Tetragonula*, *Lepidotrigona*, *Lisotrigona* and *Homotrigona* were analyzed for the first time. Based on the chemical profiles and chemometric methods, a clear separation of *H. apicalis* propolis was observed, and preference for dipterocarp tree(s) was assumed. *Tetragonula* spp. preferred the mango tree, which is one of the plants visited by *Lepidotrigona* spp. for resin collection, too. The tree *Cratoxylum cochinchinense* was shown to be a major resin source for *Lisotrigona carpenteri* propolis. Moderate to strong correlations between phenolic compounds and antioxidant capacity were found for propolis from *Tetragonula* and *Lepidotrigona* spp., as propolis from *T. laeviceps* collected in Hoa Binh and from *L. ventralis* in Quang Ning, Hoai An and Cao Bang displayed the strongest activity. The obtained results add knowledge on the chemistry and biological activity of propolis from different stingless bee species, as well as on bees’ preferences for resin sources. Further research in this direction is needed in order to show and ensure the appropriate floral resources for bees. Moreover, such studies can help to reveal possibilities for propolis standardization, which is an important prerequisite for the sustainable medical application of natural bee products.

## Figures and Tables

**Table 1:** Bee species, geographical location and time of collection of stingless bee propolis.

Code	Geographical Location	Bee Species	Time of Collection (Month/Year)
V-1	Da Nang	*Tetragonula iridipennis*	07/2018
V-2	Da Nang	*Tetragonula laeviceps*	07/2018
V-3	Hoa Binh	*Tetragonula laeviceps*	11/2018
V-4	Hoa Binh	*Tetragonula laeviceps*	11/2018
V-5	Hoa Binh	*Tetragonula laeviceps*	11/2018
V-6	Quang Ninh	*Lepidotrigona ventralis*	11/2018
V-7	Quang Ninh	*Lepidotrigona ventralis*	11/2018
V-8	Daklak	*Tetragonula laeviceps*	11/2018
V-9	Hoa Binh	*Tetragonula laeviceps*	11/2018
V-10	Hoai Xuan	*Lisotrigona carpenteri*	09/2019
V-11	Hoai Xuan	*Tetragonula laeviceps*	09/2019
V-12	Hoai Xuan	*Tetragonula iridipennis*	09/2019
V-13	Hoai Xuan	*Tetragonula laeviceps*	09/2019
V-14	Hoai Xuan	*Lepidotrigona ventralis*	09/2019
V-15	Hoai Xuan	*Homotrigona apicalis*	09/2019
V-16	Hoai Xuan	*Lepidotrigona terminata*	09/2019
V-17	Hoai Xuan	*Homotrigona apicalis*	09/2019
V-18	Hoai An	*Tetragonula iridipennis*	09/2019
V-19	Hoai An	*Tetragonula iridipennis*	09/2019
V-20	Hoai An	*Tetragonula iridipennis*	09/2019
V-21	Hoai An	*Lepidotrigona ventralis*	09/2019
V-22	Hoai An	*Tetragonula laeviceps*	09/2019
V-23	Hoai An	*Lepidotrigona terminata*	09/2019
V-24	Cao Bang	*Lepidotrigona ventralis*	06/2019
V-25	Dac Nong	*Homotrigona apicalis*	04/2019

**Table 2:** DPPH and FRAP for Vietnamese stingless bee propolis ^a^.

Sample	DPPH, %RSA	FRAP, μmol/L
*Tetragonula iridipennis*		
V-1	5.43 ± 0.69	150.63 ± 3.77
V-12	9.15 ± 0.13	149.09 ± 10.47
V-18	3.79 ± 0.18	187.90 ± 6.64
V-19	6.80 ± 0.46	246.29 ± 9.50
V-20	2.45 ± 0.11	145.01 ± 4.96
*Tetragonula laeviceps*		
V-2	9.03 ± 0.25	267.70 ± 3.33
V-3	12.71 ± 0.89	186.22 ± 2.06
V-4	46.19 ± 0.18	706.29 ± 1.70
V-5	32.21 ± 0.04	696.23 ± 8.84
V-8	9.90 ± 0.19	128.81 ± 2.19
V-9	19.09 ± 0.04	403.87 ± 3.23
V-11	6.79 ± 0.07	168.95 ± 2.14
V-13	12.26 ± 0.21	258.70 ± 15.09
V-22	34.40 ± 0.37	456.71 ± 7.49
*Lepidotrigona ventralis*		
V-6	21.93 ± 0.27	487.16 ± 4.22
V-7	35.86 ± 0.98	633.57 ± 1.10
V-14	10.36 ± 0.57	460.79 ± 3.49
V-21	66.83 ± 0.67	1013.30 ± 1.31
V-24	40.61 ± 0.33	1156.47 ± 8.12
*Lepidotrigona terminata*		
V-16	27.50 ± 0.53	415.76 ± 0.80
V-23	6.86 ± 0.37	323.52 ± 0.73
*Homotrigona apicalis*		
V-15	2.48 ± 0.14	103.58 ± 6.65
V-17	3.94 ± 0.32	117.45 ± 2.03
V-25	9.84 ± 0.41	283.30 ± 2.34
*Lisotrigona carpenteri*		
V-10	37.90 ± 0.22	367.01 ± 10.15
*Apis mellifera* ^b^ (Bulgarian propolis)	21.68 ± 0.20	410.19 ± 3.13

^a^ Tested concentration 1 mg/mL; ^b^ Tested concentration 0.1 mg/mL.

## Data Availability

The data presented in this study are available upon request from the corresponding author.

## Supplementary Materials

The following materials are available online: https://www.mdpi.com/article/10.3390/molecules27227834/s1. Table S1. Chemical composition of Vietnamese stingless bee propolis (GC-MS after silylation; %TIC); Figure S1. Loading plot and variable’s contribution in the projection (VIP) of PLS-DA model for classification of 24 propolis samples based on 43 variables in accordance with the three genera *Tetragonula*, *Lepidotrigona* and *Homotrigona*; Figure S2. Permutation tests for the three predefined classes in PLS-DA model for classification of 24 propolis samples based on 14 variables; Figure S3. ROC analysis for *Tetragonula, Lepidotrigona* and *Homotrigona* stingless bee propolis. Table S2. Misclassification table for PLS-DA model for classification of 24 propolis samples based on 14 variables of propolis in accordance with the three genera *Tetragonula, Lepidotrigona* and *Homotrigona*; Figure S4. GC chromatograms of selected samples. From bottom to top: propolis from *Tetragonula iridipennis* (V-12), *Lepidotrigona terminata* (V-16), *Homotrigona apicalis* (V-17) and *Lisotrigona carpenteri* (V-10). Table S3. Pearson correlation (*r*) between groups of phenolic constituents and DPPH and FRAP values.

## Sample Availability

Samples are available upon reasonable request from the corresponding author.
