# Supplementary material for: Chemical Profile and Antioxidant Capacity of Propolis from Tetragonula, Lepidotrigona, Lisotrigona and Homotrigona Stingless Bee Species in Vietnam
Source: Molecules. 2022 Nov 14;27(22):7834. doi: 10.3390/molecules27227834 (PMC9696581; doi:10.3390/molecules27227834)
Supplement: Supplementary file 1 [file molecules-27-07834-s001.zip › molecules-2021605-supplementary.pdf]

Supplementary material

Chemical profile and antioxidant capacity of propolis from *Tetragonula*, *Lepidotrigona*, *Lisotrigona* and *Homotrigona* stingless bee species in Vietnam

Milena Popova [1,*], Boryana Trusheva [1], Ralitsa Chimshirova [1], Daniela Antonova [1], Kamelia Gechovska [1], Le Nguyen Thanh [2,3], Nguyen Thi Phuong Lien [4], Diep Thi Lan Phuong [5] and Vassya Bankova [1]

[1] Institute of Organic Chemistry with Centre of Phytochemistry, Bulgarian Academy of Sciences, Acad. G. Bonchev, str., bl. 9, 1113 Sofia, Bulgaria

[2] Institute of Marine Biochemistry, Vietnam Academy of Science and Technology, Hanoi 10000, Vietnam

[3] Graduate University of Science and Technology, Vietnam Academy of Science and Technology, Hanoi 10000, Vietnam

[4] Institute of Ecology and Biological Resources, Vietnam Academy of Science and Technology, Hanoi 10000, Vietnam

[5] Faculty of Natural Sciences, Quy Nhon University, Binhdinh 55000, Vietnam

[*]Correspondence: milena.popova@orgchm.bas.bg

Table S1. Chemical composition of the Vietnamese stingless bee propolis [a] (GC-MS after silylation; %TIC).

**Compound**	**V-1**	**V-2**	**V-3**	**V-4**	**V-5**	**V-6**	**V-7**	**V-8**	**V-9**	**V-10**	**V-11**	**V-12**	**V-13**	**V-14**	**V-15**	**V-16**	**V-17**	**V-18**	**V-19**	**V-20**	**V-21**	**V-22**	**V-23**	**V-24**	**V-25**
	Bee species																								
	T.i.	*T.l.*	*T.l.*	*T.l.*	*T.l.*	*L.v.*	*L.v.*	*T.l.*	*T.l.*	*L.c.*	*T.l.*	*T.i.*	*T.l.*	*L.v.*	*H.a.*	*L.t.*	*H.a.*	*T.i.*	*T.i.*	*T.i.*	*L.v.*	*T.l.*	*L.t.*	*L.v.*	*H.a.*
**Sugars and sugar alcohols**	**17.4**	**20.9**	**11.3**	**15.1**	**10.4**	**10.0**	**22.4**	**16.7**	**16.1**	**7.7**	**12.9**	**12.3**	**12.8**	**12.1**	**10.6**	**19.7**	**15.8**	**15.6**	**13.9**	**20.2**	**20.3**	**18.8**	**18.2**	**11.6**	**12.1**
**Fatty acids**	**0.8**	**0**	**0.2**	**0**	**0**	**0.3**	**0**	**0.2**	**0**	**0**	**0.1**	**0.2**	**0.1**	**2.5**	**1.2**	**0.3**	**1.1**	**0.2**	**0.3**	**0.1**	**0.1**	**0.2**	**0.4**	**0.7**	**0.2**
Hexadecanoic acid	0.5	Tr.	0.1	Tr.	0	0.3	0.1	0	0	0	0.1	0.1	0.1	2.5	0.3	0.2	0.6	0.1	0.2	0.1	Tr.	0.2	0.3	0.6	0.2
Octadecanoic acid	0.2	0	0.1	0	Tr.	0	0	0.2	0	Tr.	0	0.1	0	0	0.5	0.1	0.5	0.1	0.1	0	0.1	0	0.1	0.1	Tr.
Eicosanoic acid	0.1	0	0	0	0	0	0.1	0	0	0	0	0	0	0	0.4	0	0	0	0	0	0	0	0	0	0
**Aromatic acids**	**0**	**0**	**0**	**0**	**0**	**0.6**	**0.8**	**0**	**0**	**1.9**	**0**	**0.5**	**0.1**	**3.7**	**0.1**	**0.4**	**0**	**0**	**0.2**	**0.1**	**14.5**	**0.3**	**0.3**	**37.2**	**0.4**
Benzoic acid	0	0	0	0	0	Tr.	Tr.	0	0	0.8	0	0	0	0.2	0	Tr.	0	0	0	0.1	7.1	0.1	0.1	27.5	0.2
p-Hydroxybenzoic acid	0	0	0	0	0	0.1	0.3	0	0	0.3	0	0.3	0.1	1.0	0.1	0	0	0	0.2	0	0.5	0	0.1	0.2	0.2
Protocatechuic acid	0	0	0	0	0	0.3	0.2	0	0	0.4	0	0.1	0	0	0	0.1	0	0	0	0	5.4	0.2	0.1	4.3	0
Cinnamic acid	0	0	0	0	0	0	0.2	0	0	0	0	0.1	0	1.0	0	0.1	0	0	0	0	0.1	0	0	2.0	0
Vanillic acid	0	0	0	0	0	0.2	0.1	0	0	0.4	0	0	0	1.5	0	0.2	0	0	0	0	1.4	0	Tr.	3.2	0
**Cardanols**	**0.9**	**4.4**	**2.1**	**7.4**	**0.6**	**0.4**	**0.1**	**5.5**	**1.4**	**0**	**1.0**	**1.1**	**6.3**	**0.2**	**0**	**0.1**	**0**	**0.1**	**0.3**	**0.1**	**0**	**0.2**	**0**	**0.2**	**0**
Cardanol C13:0	0	0.2	0	0	0	0	0	0	0	0	0	0	0	0	0	0	0	0	0	0	0	0	0	0	0

Cardanol C13:1	0	0.5	0	0	0	0	0	0	0	0	0	0	0	0	0	0	0	0	0	0	0	0	0	0	0
Cardanol C15:0	0	0.4	0.1	3.5	0.3	0	0	0.2	0.1	0	0.1	0.1	0.3	Tr.	0	0	0	0	0	0	0	0	0	0.1	0
Cardanol C15:1	0	1.8	0.2	3.9	0.3	0	Tr.	0.3	0.3	0	0	0	0.3	0	0	0	0	0	0.1	0	0	0	0	0.1	0
Cardanol C17:0	0.1	0	0.1	0	0	0.3	0	0.4	0	0	0.1	0.1	0.7	0.1	0	0	0	0	0	0	0	0	0	0	0
Cardanol C17:1	0.4	0	0.9	0	0	0	0.1	2.4	0.4	0	0.4	0.6	3.3	0.1	0	0.1	0	0.1	0.2	0.1	0	0.2	0	0	0
Cardanol C17:3	0	0.4	0	0	0	0	0	0.4	0	0	0	0	0	0	0	0	0	0	0	0	0	0	0	0	0
Cardanol C19:1	0.4	1.1	0.8	0	0	0.4	0	1.8	0.6	0	0.4	0.3	1.7	0	0	0	0	0	0	0	0	0	0	0	0
**Resorcinols (cardols)**	**7.2**	**26.4**	**34.2**	**1.3**	**1.2**	**17.7**	**1.3**	**34.1**	**20.4**	**0**	**7.8**	**13.5**	**37.1**	**13.2**	**0**	**2.1**	**0**	**4.1**	**0.6**	**0.4**	**0.1**	**0.2**	**2.1**	**0**	**0**
Resorcinol C15:0	0.4	1.6	1.5	0	Tr.	0	0	1.1	0.9	0	0.4	0.6	1.4	1.2	0	0.2	0	0.2	0	Tr.	0	0	0.1	0	0
Resorcinol C15:1	0.2	3	2.3	0	0	0	0	1.3	1.1	0	0.4	0.4	1.3	4.4	0	0.2	0	0.1	0	0	0	0	0.1	0	0
Resorcinol C17:0	0.5	2.6	0	1.3	0.2	1	0.4	2	1.5	0	0.2	0.7	1.6	0	0	0.1	0	0.2	0	0	Tr.	0.1	0.1	0	0
Resorcinols C17:1	4.5	12.1	22.5	0	0	12.3	0	22.4	10.8	0	5.7	10.7	25.7	6.8	0	1.1	0	2.6	0.6	0.4	0.1	0.1	1.6	7r.	0
Resorcinols C17:3	1.6	2.6	0.8	0	0	0.5	0	1.1	0.6	0	0	0	1.8	0	0	0	0	0.2	0	0	0	0	0	0	0
Resorcinol C19:0	0	4.5	0.5	0	0	0	0	0.2	0.2	0	0	0	0.1	0	0	0	0	0	0	0	0	0	0	0	0
Resorcinols C19:1	0	0	6.6	0	1	3.9	0.9	6	5.3	0	1.1	1.1	5.2	0.8	0	0.5	0	0.8	0	0	0	0	0.2	0	0
**Anacardic acids**	**3.7**	**7.1**	**16.2**	**1.6**	**4.3**	**3.5**	**2.6**	**7.3**	**15.4**	**0**	**2.7**	**8.7**	**9**	**0.5**	**0**	**0.4**	**0**	**0.9**	**0.3**	**0.7**	**0.1**	**0.2**	**0.2**	**0.1**	**0**
Anacardic acid C15:0	0.1	0	3	1.6	0	0	0	0	Tr.	0	0	0	0	0.1	0	Tr.	0	0	0	0	Tr.	0.1	0	Tr.	0
Anacardic acids C15:1	0.9	0.5	1	0	3.4	0.4	1.9	0	5.8	0	0	0.5	0	0.1	0	0	0	0.1	0	0	0	0	0	0	0
Anacardic acids C17:1	0	3.6	7.1	0	0.5	3.1	0.3	3.8	4.5	0	1.3	4.8	4.2	0.3	0	0.4	0	0	0.2	0.2	0.1	0.1	0.2	0.1	0
Anacardic acid C17:3	0	0	0.2	0	0	0	0	0	0	0	0	0	0	0	0	0	0	0	0	0	0	0	0	0	0

Anacardic acid C19:0	0	0	0	0	0	0	0	0.2	0	0	0.7	0.2	2.4	0	0	0	0	0.4	0.1	0	0	0	0	0	0
Anacardic acid C19:1	2.7	3	4.9	0	0.4	0	0.4	3.3	5.1	0	0.7	3.2	2.4	0	0	0	0	0.4	0	0.5	0	0	0	0	0
**Triterpenes**	**35.6**	**22.8**	**28.9**	**33.0**	**28.9**	**32.2**	**28.9**	**26.1**	**33.1**	**14.3**	**31.6**	**31.9**	**18.4**	**22**	**50.8**	**39.3**	**45.6**	**33.6**	**28.8**	**24**	**28.2**	**20.5**	**31.6**	**16.4**	**56.2**
(13*E*,17*E*)-Polydopa-7,13,17,21-tetraen-3*β*-ol [b]	0	0	0.1	0	0.8	0	0	0	0	11.2	0	0	0	0	0	0.3	0	0	0	0	0	0.6	0	0	0
Lanosterol[c]	1.8	2	0.2	0	0	0.5	0.6	0.3	0.9	0	0	0	0.4	2.9	0.4	0.8	0	0.4	0.2	0	0	0	0	0	0
Lanosterol (3-*epi*)[c]	3	2.7	0	0	0	0	0	0.4	0.2	0	0	0.6	0	3.1	0	0.3	0	0.6	0	0	0	0	1	0	0
α-Amyrine	3.3	0	2.7	0.8	1.5	2.8	2.9	1.7	3.2	0.9	2.8	2.5	2.1	2.8	6.8	4.5	1.2	7.4	1.1	0	5.6	3.9	0	2	0.7
β-Amyrine	5.2	2.2	2.2	0.9	1.0	3.2	3.8	1.4	2.7	0.4	4.4	3.0	2.4	4.3	9.0	9.5	2.1	3.3	0	5.1	8.7	2.6	7.2	1.4	0.5
α-Amyrenone	2.2	0	0	0	0	0.7	0.9	0.5	0	0	3.0	0	0	0	0	1.7	0	0	2.1	4.5	3.9	3.4	3.1	0	0
β-Amyrenone	3.1	0	0	0	0	0.7	1.2	0.6	0.1	0	1.1	0	0	0	0	1	0	0	0	2.8	2.9	0	1.6	0.9	0
Ursolic acid	0	0	0	1	3.2	2.1	2.8	0	0.8	0	0	0	0	0	4.9	0	0	0	0	0	0	0	0	0	0
Oleanolic acid	0	0	0	0	0	0	0.4	0	0	0	0	0	0	0	1.9	0	0	0	0	0	0	0	0	0	0.1
Ursonic acid	0	0	0	2.7	2.7	2.9	2.5	0	0	0	0	0	0	0	4.9	0	1	1.9	0	0	0	0	0	2.7	3.8
Moronic acid	0	0	0	3.5	2.4	0.9	1.1	0	0	0	0	0	0	0	0	0	0	0	0	0	0	0	0	0	0
Oleanonic acid	0.1	0	0	0	0	0	0.2	0	0	0	0	0	0	0	1.9	1	2.6	1.3	0	0	0	0	0	1.8	1.9
Lantanolic acid[b]	0	0	1.2	11.5	3.4	0	2.8	0	1.5	0	0	0	0	0	0	0	0	0	0	0	0	0	0	0	0
Uvaol	0	0	0	0	0	0	0	0	0	0	0	0	0	0	1.5	0	0.4	0	0	0	0	0	0	0	1.1
Erythrodiol	0	0	0	0	0	0	0	0	0	0	0	0	0	0	0.2	0	0.8	0	0	0	0	0	0	0	0.6
Ursolic aldehyde	0	0	0	0.1	0	0	0	0	0	0	0	0	0	0	3.0	1.1	3.2	0	0	0	0	0	0	0	2.2
Oleanolic aldehyde	0	0	0	0	0	0	0	0	0	0	0	0	0	0	4.6	0	8.8	0	0	0	0	0	0	0	3.4

Ursolic acid acetate	0	0	0	0	0	0	0	0	0	0	0	0	0	0	1.1	0	2.2	0	0	0	0	0	0	0	1.7
Oleanolic acid acetate	0	0	0	0	0	0	0	0	0	0	0	0	0	0	2.2	0	5.7	0	0	0	0	0	0	0	6.2
Triterpene acid, amyrine type	0	0	0	0	0	0	0	0	0	0	0	0	0	0	3.8	0	15.3	3.3	9.2	0	0	0	0	0	16.9
(M^+= 686, base peak 203)																									
Cycloartenol	6.3	4.9	3.4	0	0.8	1.9	1.4	3.2	2.9	0	3.8	5.2	5.3	0	0	6.1	0	4.1	3.5	0.8	1	0.1	3.2	0.4	0
24-Methylenecycloartenol	0	0	0.1	0	0	0	0	0	0	0	1.2	0	1	0	0	0	0	0	0	0	1.5	0	0	0	0
Cycloartenone	3	1.2	0	0	0	1.0	0	1.2	0.9	0	3.8	5.2	5.3	0	0	6.1	0	4.1	4.0	0.8	1	0	3.2	0.4	0
Isomangiferolic acid[c]	0.1	3.8	2.2	0.4	0.4	2.4	0.3	3.8	2.9	0	6.9	9.9	0.8	0.7	0	2.0	0	1.5	1.7	0	0	0.1	2.0	0	0
Mangiferolic acid[c]	0.1	1.6	10.1	0.2	3.0	6.0	1.0	6.2	3.4	0	0.1	1.2	0.2	5.0	0	0.3	0	0.2	0.1	0	0	0	0	0	0
Ambolic acid	0	0	2.2	0	0	0.8	0.9	3.6	8.6	0	0	0	0	0	0	0	0	0	0	0	0	0	0	0	0
24-Hydroxyisomangiferolic acid	0	0	0.4	0	0	0	0	0	1.4	0.9	0	0	0	0	0	0	0	0	0	0	0	0	0	0	0
Lupeol	3.1	2.7	1.8	0.5	0.9	2.9	3.4	1.7	2	0.9	2.1	3.9	0.9	3.2	1.5	3.8	0.5	1.3	3.1	1.1	3.6	6.9	0	5.1	0.7
Lupenon	0	1.7	0.3	0	0	0	0	0.4	0	0	2.5	1.6	0	0	0	0	0	0	0	0	0	2.8	1.1	1.7	0
Betulinic acid	1.9	0	0	2.1	1.2	0	0.7	0.2	0.6	0	0	0	0	0	1.9	0	0	0	0	0	0	0	0	0	0
Betulonic acid	0	0	0	6.9	3.1	0	0.4	0	0.6	0	0	0	0	0	0	0	0	0	0	0	0	0	0	0	0
Dammarenon	0	0	0	0.1	0.1	0	0.2	0	0	0	0	0	0	0	0	0.8	0	2	0	3.3	0	0	6.8	0	0
Dammarenol	0	0	0	0	0	0	0	0	0	0	0	0	0	0	0.3	0	1.8	0	0	0	0	0	0	0	6.3
Dipterocarpol	0	0	0	0	0	0	0	0	0	0	0	0	0	0	0	0.9	0	2.1	3.8	5.6	0	0	2.3	0	10.1
Triterpene alcohol (M^+=498)	0	0	2.0	0	0	1.7	1.4	0.9	1.3	0	0	0	0	0	0	0	0	0	0	0	0	0	0	0	0

Triterpene hydroxy acid (M^+=598)	2.4	0	0	1.9	1.4	1.7	0	0	0	0	0	0	0	0	0	0	0	0	0	0	0	0	0	0	0
Triterpenic acid (M^+=616)	0	0	0	0.4	3.0	0	0	0	0	0	0	0	0	0	0	0	0	0.1	0	0	0	0.1	0	0	0
**Xanthones**	**0**	**0**	**4.8**	**28.9**	**23.3**	**1.5**	**6.5**	**0**	**2.5**	**47**	**0**	**0**	**0**	**20.5**	**0**	**2.8**	**0**	**0**	**0**	**0**	**8.1**	**38.6**	**0**	**0**	**0**
4-Geranyl-1,3,5-trihydroxyxanthone[b]	0	0	0.7	4.2	2.7	0	0.6	0	0	0	0	0	0	0	0	0	0	0	0	0	0	0	0	0	0
α-Mangostin[b]	0	0	1.4	4	2.7	0.9	1.4	0	0.5	7.6	0	0	0	4.6	0	0.5	0	0	0	0	0	6.3	0	0	0
Dihydromangostanine[b]	0	0	0.8	0	4.1	0	1.7	0	1	0.3	0	0	0	0	0	0	0	0	0	0	3.0	0	0	0	0
Cochinchinone A[b]	0	0	1.1	15.8	7.1	0.6	1.7	0	1	36.3	0	0	0	4.2	0	0.5	0	0	0	0	4.1	20.7	0	0	0
Cratoxylumxanthone B[b]	0	0	0	0	0	0	0	0	0	0.2	0	0	0	0.3	0	0	0	0	0	0	0	4.7	0	0	0
Cochinchinone K[b]	0	0	0.8	3.5	3.6	0	0.7	0	0	2.6	0	0	0	1.7	0	0	0	0	0	0	1.0	6.9	0	0	0
Xanthone (M^+=536)	0	0	0	0	0	0	0	0	0	0	0	0	0	9.7	0	1.8	0	0	0	0	0	0	0	0	0
Xanthone (M^+=554)	0	0	0	1.4	3.1	0	0.4	0	0	0	0	0	0	0	0	0	0	0	0	0	0	0	0	0	0
**Tocotrienols**	**0**	**0**	**0**	**0.2**	**0.3**	**0**	**0**	**0**	**0**	**1.8**	**0**	**0**	**0**	**0.8**	**0**	**0.1**	**0**	**0**	**0**	**0**	**0**	**1.1**	**0**	**0**	**0**
d-Tocotrienol	0	0	Tr.	0.2	0.2	0	Tr.	0	0	0.9	0	0	0	0.6	0	0.1	0	0	0	0	Tr.	0.8	0	0	0
β/γ-Tocotrienol	0	0	0	0	0.1	0	0	0	0	0.9	0	0	0	0.2	0	0	0	0	0	0	0	0.3	0	0	0
**Unknowns**	**0**	**0**	**0**	**0**	**0**	**0**	**0**	**0**	**0**	**0**	**0**	**0**	**0**	**0**	**0**	**0**	**0**	**11.2**	**19.7**	**6.0**	**0**	**0**	**0**	**0**	**0**
Unknown 1	0	0	0	0	0	0	0	0	0	0	0	0	0	0	0	0	0	5.7	8.1	0	0	0	0	0	0
Unknown 2	0	0	0	0	0	0	0	0	0	0	0	0	0	0	0	0	0	2.2	5.6	0	0	0	0	0	0
Unknown 3	0	0	0	0	0	0	0	0	0	0	0	0	0	0	0	0	0	1.5	5.7	2.4	0	0	0	0	0

Unknown 4	0	0	0	0	0	0	0	0	0	0	0	0	0	0	0	0	0	1.8	5.9	3.6	0	0	0	0	0

[a] The detailed information (bee species, geographical location and time of collection) for the propolis samples from V-1 to V-25 is presented in Table 1; [b] Indentified by reference compounds; [c] Intercheangable compounds. *T.i.* – *Tetragonula iridipennis*; *L.v.*- *Lepidotrigona ventralis*; *T.l.* – *Tetragonula laeviceps*; *L.c.* – *Lisotrigona carpenteri; H.a.* – *Homotrigona apicalis*; *L.t.* – *Lepidotrigona terminata*. Tr. – traces (< 0.05 %TIC).

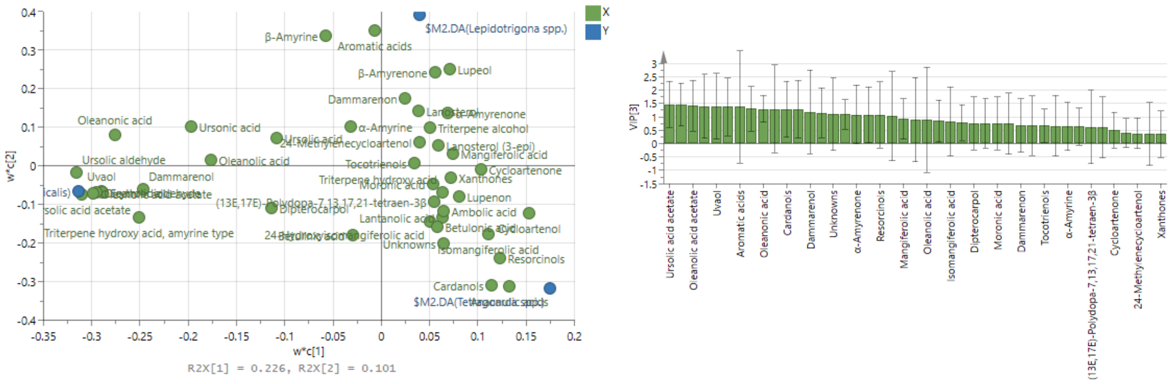

Figure S1. Loading plot and variable's contribution in the projection (VIP) of PLS-DA model for classification of 24 propolis samples based on 43 variables in accordance with the three stingless bee genera *Tetragonula*, *Lepidotrigona* and *Homotrigona*.

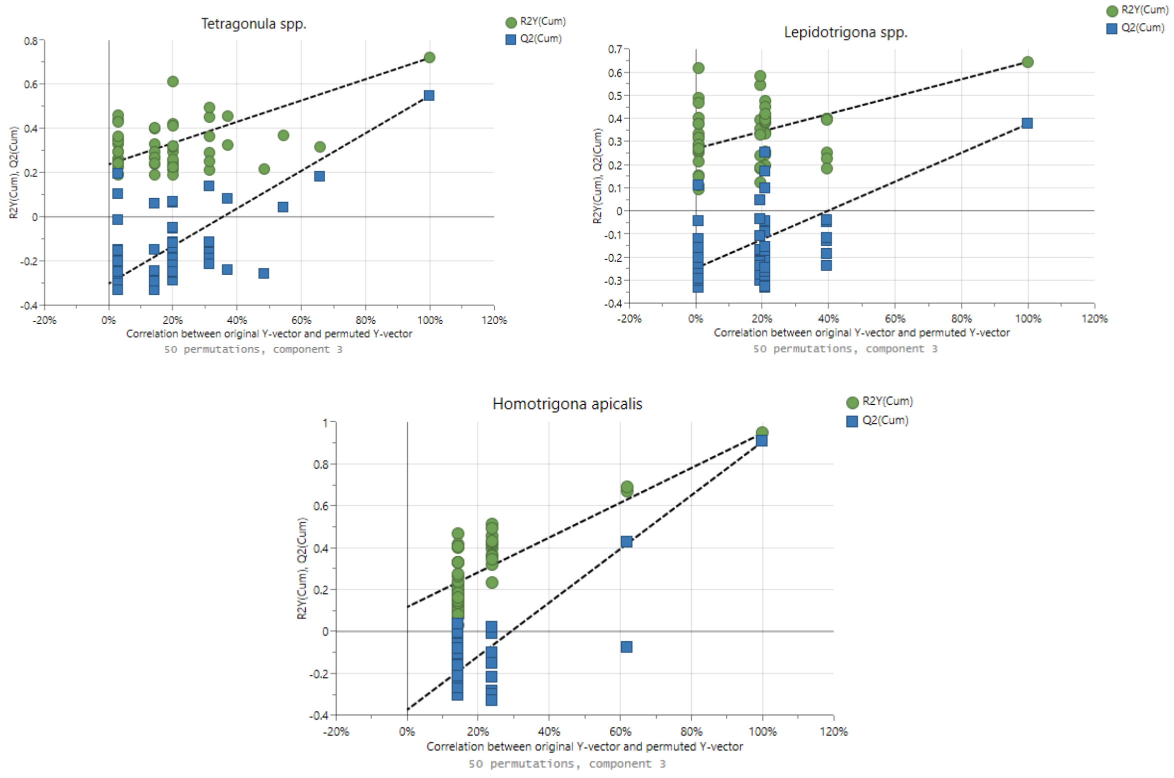

Figure S2. Permutation tests for the three predefined classes in PLS-DA model for classification of 24 propolis samples based on 14 variables.

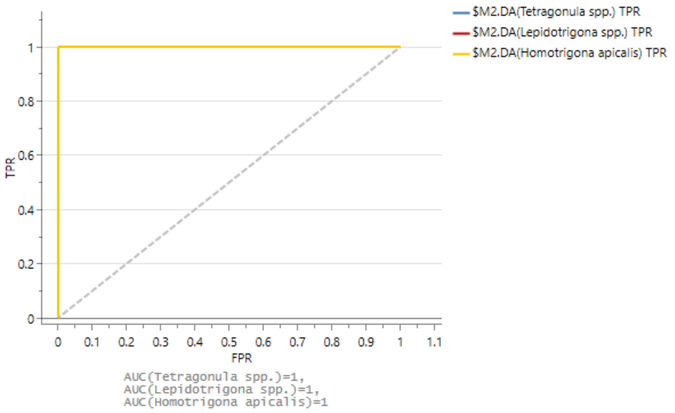

Figure S3. ROC analysis for *Tetragonula*, *Lepidotrigona* and *Homotrigona* stingless bee propolis.

Table S2. Misclassification table for PLS-DA model for classification of 24 propolis samples based on 14 variables propolis in accordance with the three genera *Tetragonula*, *Lepidotrigona* and *Homotrigona*.

	**Members**	**Correct**	***Tetragonula* spp.**	***Lepidotrigona* spp.**	***Homotrigona apicalis***
***Tetragonula* spp.**	14	100%	14	0	0
***Lepidotrigona* spp.**	7	85.71%	1	6	0
Homotrigona apicalis	3	100%	0	0	3
Total	24	95.83%	15	6	3
Fisher's prob.	6.4e-08				

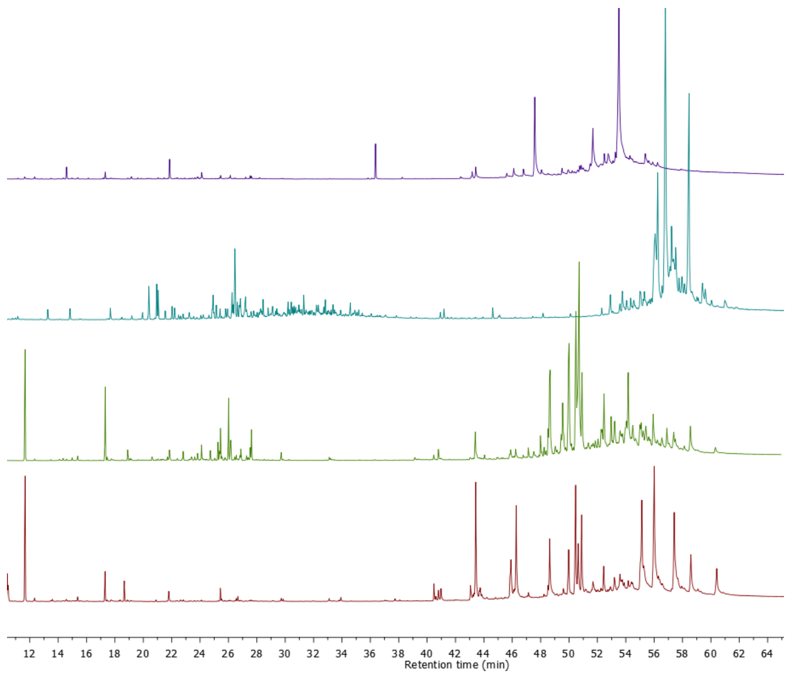

Figure S4. GC chromatograms of selected samples. From bottom to top: propolis from *Tetragonula iridipennis* (V-12), *Lepidotrigona terminata* (V-16), *Homotrigona apicalis* (V-17) and *Lisotrigona carpenteri* (V-10).

Table S3. Pearson correlation (*r*) between groups of phenolic constituents and DPPH and FRAP values.

Correlation among variables	DPPH	FRAP
Phenolic acids	0.623**	0.786**
Cardanols	0.032	-0.064
Resorcinols	-0.287	-0.313
Anacardic acids	-0.175	-0.231
Xanthones	0.538**	0.302
Phenolic acids + cardanols + resorcinols + anacardic acids + xanthones	0.220	0.315
Phenolic acids + xanthones	0.624**	0.417*

*Correlation is significant at the 0.05 level (two–tailed). ** Correlation is significant at the 0.01 level (two–tailed).
